# Combined Hypothermic and Normothermic Machine Perfusion Improves Functional Recovery of Extended Criteria Donor Livers

**DOI:** 10.1002/lt.25315

**Published:** 2018-12-04

**Authors:** Yuri L. Boteon, Richard W. Laing, Andrea Schlegel, Lorraine Wallace, Amanda Smith, Joseph Attard, Ricky H. Bhogal, Desley A. H. Neil, Stefan Hübscher, M. Thamara P. R. Perera, Darius F. Mirza, Simon C. Afford, Hynek Mergental

**Affiliations:** ^1^ Liver Unit; ^2^ Department of Pathology Queen Elizabeth Hospital, University Hospitals Birmingham National Health Service Foundation Trust Birmingham United Kingdom; ^3^ National Institute for Health Research, Birmingham Biomedical Research Centre, Institute of Immunology and Immunotherapy, College of Medical and Dental Sciences University of Birmingham Birmingham United Kingdom

## Abstract

Hypothermic oxygenated perfusion (HOPE) and normothermic perfusion are seen as distinct techniques of ex situ machine perfusion of the liver. We aimed to demonstrate the feasibility of combining both techniques and whether it would improve functional parameters of donor livers into transplant standards. Ten discarded human donor livers had either 6 hours of normothermic perfusion (n = 5) or 2 hours of HOPE followed by 4 hours of normothermic perfusion (n = 5). Liver function was assessed according to our viability criteria; markers of tissue injury and hepatic metabolic activity were compared between groups. Donor characteristics were comparable. During the hypothermic perfusion phase, livers down‐regulated mitochondrial respiration (oxygen uptake, ***P*** = 0.04; partial pressure of carbon dioxide perfusate, ***P*** = 0.04) and increased adenosine triphosphate levels 1.8‐fold. Following normothermic perfusion, those organs achieved lower tissue expression of markers of oxidative injury (4‐hydroxynonenal, ***P*** = 0.008; CD14 expression, ***P*** = 0.008) and inflammation (CD11b, ***P*** = 0.02; vascular cell adhesion molecule 1, ***P*** = 0.05) compared with livers that had normothermic perfusion alone. All livers in the combined group achieved viability criteria, whereas 40% (2/5) in the normothermic group failed (***P*** = 0.22). In conclusion, this study suggests that a combined protocol of hypothermic oxygenated and normothermic perfusions might attenuate oxidative stress, tissue inflammation, and improve metabolic recovery of the highest‐risk donor livers compared with normothermic perfusion alone.

**Abbreviations**
4‐HNE: 4‐hydroxynonenal
ALT: alanine aminotransferase
ATP: adenosine triphosphate
CD: cluster of differentiation
CIT: cold ischemia time
DAMP: damage‐associated molecular pattern
DBD: donation after brain death
DCD: donation after circulatory death
DRI: donor risk index
ECD: extended criteria donor
ET: Eurotransplant
HA: hepatic artery
HBD: hypoxic brain damage
HOPE: hypothermic oxygenated perfusion
HTN: hypertension
ICH: intracranial hemorrhage
IQR: interquartile range
IRS: immunoreactive score
NA: not applicable
NHS: National Health Service
NIHR: National Institute for Health Research
NMP: normothermic machine perfusion
PAS: periodic acid–Schiff
PCO_2_: partial pressure of carbon dioxide
PV: portal vein
ROS: reactive oxygen species
SCS: static cold storage
UCP‐2: uncoupling protein 2
VCAM1: vascular cell adhesion molecule 1
WIT: warm ischemia time

The rising incidence of liver disease, in combination with changes in organ donor demographics, has increased reliance on extended criteria donor (ECD) livers for transplantation globally.^1^, ^2^ Although these organs have inferior outcomes compared with standard criteria livers, their use is deemed necessary to control the wait‐list mortality.^3^, ^4^, ^5^ Current utilization of ECD livers remains relatively low mainly when risk factors concur. For example, in the United Kingdom, 17% of the procured livers are currently not transplanted.^6^

Ex situ machine perfusion is a novel preservation method developed to protect organs from the detrimental effects of ischemia during static cold storage (SCS). Potential beneficial protective mechanisms of machine perfusion have been demonstrated for both hypothermic and normothermic perfusion techniques during preclinical experiments and pilot clinical studies.^7^, ^8^, ^9^

Hypothermic oxygenated perfusion (HOPE) may permit mitochondrial functional recovery, increasing cellular adenosine triphosphate (ATP) levels, and mitigate the injury to the tissue that occurs during rewarming.^9^ Although there is mounting evidence of the protective effect of HOPE on the liver graft quality, evidence regarding its value in viability testing is still under investigation. Previous studies reported a correlation between perfusate transaminases content and its posttransplant levels, suggesting that hepatocellular injury could potentially be assessed also outside of normothermia.^10^, ^11^ Normothermic machine perfusion (NMP) of the liver enables metabolism at physiological temperature and, therefore, facilitates functional assessment.^12^ Several groups including our own have demonstrated that viability assessment by NMP can be used to select transplantable livers from the pool of currently discarded organs.^12^, ^13^ There are no convincing data, however, to show that NMP improves the quality of ECD organs injured by cold ischemia storage. The key benefit of NMP might be in preventing any deterioration of liver quality from the time of commencing the perfusion and by providing a snapshot of the organ injury occurred, with the opportunity to assess multiple functional parameters.

Data from our research on NMP perfusions showed that a proportion of poor‐quality livers exposed to prolonged cold storage do not recover their function and fail our viability criteria (unpublished observations). Although hypothermic perfusion and normothermic perfusion were developed as distinct strategies, we hypothesized that HOPE might be seen as a beneficial therapeutic intervention by restoring liver metabolism prior to a period of normothermic perfusion, which permits liver viability testing. The present study aimed to assess the feasibility of a protocol combining HOPE with NMP and to investigate its potential benefits over NMP alone.

## Patients and Methods

### Study Design

The study was designed to compare 2 perfusion strategies to restore function of high‐risk ECD livers, following a period of SCS, within the certified 6‐hour timeframe allowed by the used perfusion device.

**Figure 1:**
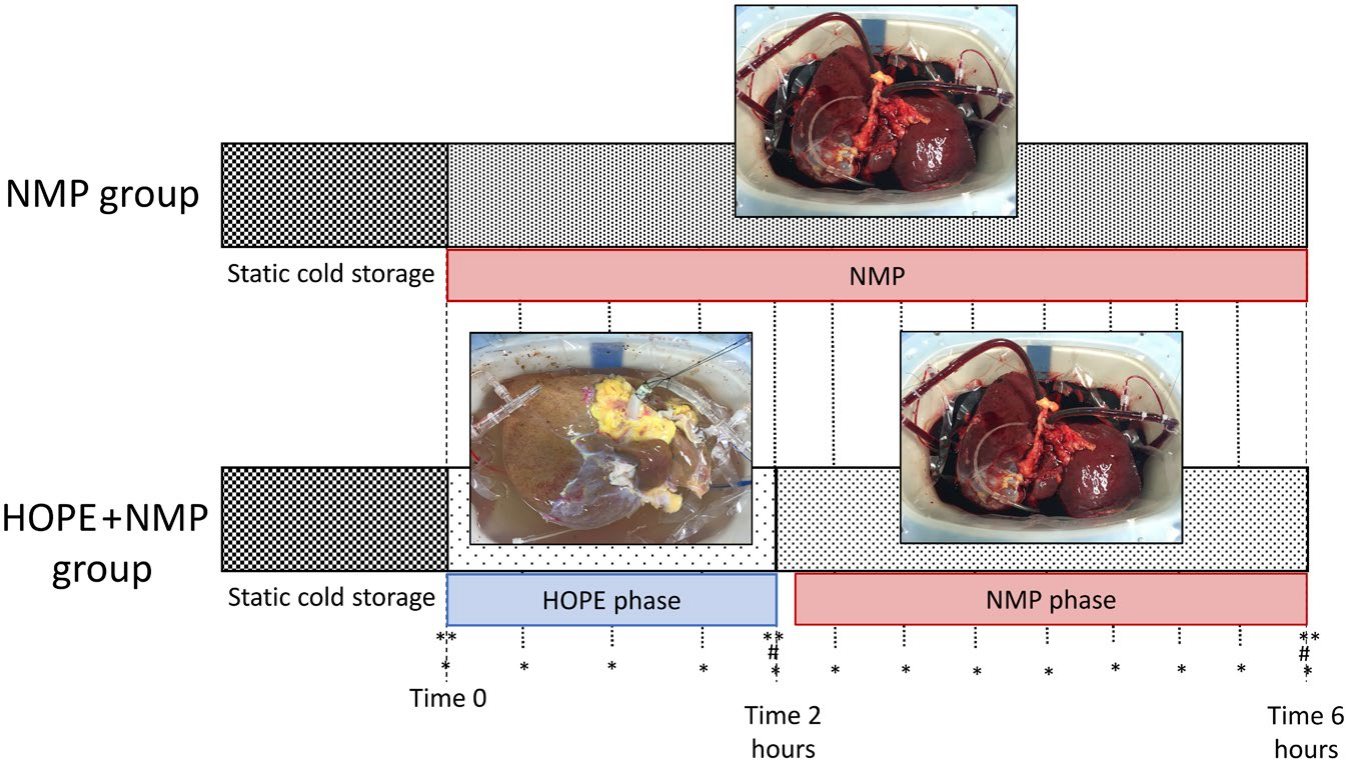
Study design. Discarded human livers were subjected to our routine organ procurement procedure and then cold flushed and cold stored. The organs were allocated randomly into 2 experimental groups of end‐ischemic machine perfusion. The NMP group was subjected to 6 hours NMP at 37°C, and the HOPE + NMP group was subjected to 2 hours of HOPE followed by 4 hours of NMP. Menghini and wedge biopsies were collected at time 0 and 6 hours (**) and immediately fixed in formalin or snap‐frozen in liquid nitrogen. The HOPE + NMP group had an extra liver biopsy taken at 2 hours. Blood gas analysis was carried out, and perfusate was sampled at 30‐minute time intervals throughout (*). In addition, bile was collected and weighed at a time of 4 and 6 hours (#).

The study endpoints were evaluation of hepatocellular injury and liver function assessment. Ten discarded human donor livers were consecutively assigned to 2 study groups, each consisting of 5 organs and all perfused for 6 hours. Livers in 1 group were exposed to normothermic perfusion alone (NMP group, performed first), while the other group underwent 2 hours of HOPE, followed by 4 hours of normothermic perfusion (HOPE + NMP group). Figure 1 shows the study design and sampling protocol.

### Source of Discarded Human Livers

All included livers were procured with the intention of transplantation according to the National Organ Retrieval Service standards. The organs were declined for clinical use by all the UK liver transplant centers and subsequently offered for research purposes. The livers were preserved in University of Wisconsin fluid under standard clinical practice of SCS prior to commencing perfusion. The study protocol was approved by the appropriate institutional review committee. Ethical approval for the study was obtained by the London‐Surrey Borders National Research Ethics Service and the National Health Service Blood and Transplant Ethics Committee (references 13/LO/1928 and 06/Q702/61, respectively).

### Liver Perfusion Procedure

The liver preparation for the perfusion was carried out using a standard clinical back‐table procedure as described elsewhere.^14^ The cystic duct was ligated, and the celiac trunk, portal vein (PV), and bile duct were cannulated with a 12‐Fr biliary drain. Before commencing machine perfusion, the liver was flushed with 2 L of 5% glucose solution and placed into the device reservoir. Then the cannulas were connected to the perfusion circuits. The Liver Assist device (Organ Assist, Groningen, the Netherlands) provides dual perfusion of the hepatic artery (HA) and PV in a semiclosed circuit by 2 rotary pumps to produce pulsatile and nonpulsatile flows, respectively.^15^ The perfusate temperature and perfusion pressures were set by the operator, and the measured flow rates and calculated resistances, shown on the device’s display in real time, were recorded every 30 minutes. Oxygen was supplied via a Sechrist air/oxygen blender (S3500CP‐G, Inspiration Healthcare, Ltd., Leicester, United Kingdom) with the fraction of inspired oxygen and air flow adjusted as specified below.

### Hypothermic Oxygenated Perfusion

HOPE was performed via PV only, using 3 L of Belzer MPS University of Wisconsin Machine Perfusion Solution (Bridge to Life, Ltd., EU) with the temperature set at 10°C. The target flow was 0.1 mL/minute/g of liver with a maximum pressure of 3 mm Hg. The target oxygen perfusate pressure was 80‐100 kPa. After 2 hours of HOPE, the perfusion was stopped and the liver temporarily placed on ice. The system was then drained and subsequently refilled with the NMP perfusion solution. The HA and common bile duct were cannulated and then the normothermic perfusion commenced. The perfusate exchange, from the end of HOPE to the start of NMP, took an average of 20 minutes.

### Normothermic Machine Perfusion

The perfusion fluid for NMP consisted of 1000 mL (4 units) of an acellular, polymerized bovine hemoglobin‐based oxygen carrier Hemopure (Hemoglobin Oxygen Therapeutics LLC, Cambridge, MA) complemented with human albumin solution and additional supplements as described in Supporting Table 1. For superior oxygen delivery, rheological characteristics, and research logistics, this solution became our preferred perfusate.^16^

**Table 1:** Donor Demographics, Liver Characteristics, and Machine Perfusion Parameters

	NMP 1	NMP 2	NMP 3	NMP 4	NMP 5	HOPE + NMP 1	HOPE + NMP 2	HOPE + NMP 3	HOPE + NMP 4	HOPE + NMP 5
Donor information										
Age, years	70	36	50	25	60	54	50	54	38	55
Donor type	DCD	DCD	DBD	DCD	DCD	DCD	DBD	DBD	DCD	DCD
Sex	Male	Male	Male	Male	Male	Male	Female	Female	Male	Female
Height, cm	165	181	187	175	189	179	158	170	183	160
Body weight, kg	80	70	90	82	75	123	60	87	85	90
Body mass index, kg/m^2^	29	21	25	26	21	38	24	30	25	35
DRI										
United States	3.2	2.6	1.6	2.2	2.2	2.5	2.0	1.9	1.8	3.0
United Kingdom	4.0	2.1	0.9	2.1	27.4	16.3	6.9	1.2	3.1	4.9
ET	3.3	2.2	1.7	1.9	2.4	2.9	2.2	1.9	2.3	2.8
Peak ALT, IU/L	168	17	44	476	137	189	14	271	37	741
Days on ventilator	1	5	1	3	8	5	2	2	4	4
Comorbidities and risk history	HTN	Smoker	Alcohol misuse	Smoker, alcohol misuse	Smoker	Diabetes (type 2) HTN	Smoker, alcohol misuse	Diabetes (type 1), smoker	Active smoker	HTN
Cause of death	ICH	ICH	ICH	Trauma	Trauma	HBD	ICH	ICH	ICH	HBD
Liver characteristics										
Liver weight, g	2208	2218	2380	1998	1555	2600	1838	2060	1753	1935
Donor WIT, minutes	24	20	NA	10	17	31	NA	NA	42	8
CIT, minutes	384	453	464	612	446	497	682	491	660	510
Retrieval location	Regional	Extrazonal	Extrazonal	Extrazonal	Extrazonal	Extrazonal	Extrazonal	Extrazonal	Extrazonal	Extrazonal
Reason for clinical rejection	Steatosis	Poor flush	Donor cancer	High ALT, poor flush	Donor cancer	Poor flush	Steatosis	Steatosis	Poor flush	Steatosis, poor flush
Large‐droplet macrovesicular steatosis (paraffin sections)	60%	0%	0%	0%	0%	10%	0%	15%	3%	30%
Machine perfusion parameters										
Lactate, mmol/L										
Highest	9.6	20.0	10.3	10.4	9.0	9.1	8.9	10.4	4.6	7.8
Lowest	4.1	8.8	0.3	1.4	0.6	0.6	1.1	1.8	0.2	0.8
At the end of 6‐hour perfusion	5.3	11.6	0.3	1.6	1.4	0.6	1.1	1.8	0.2	0.8
Total bile production, g	0.0	0.0	17.6	26.0	38.0	57.0	0.0	0.0	15.9	24
Mean arterial flow, mL/minute	535	256	760	529	616	292	299	234	152	398
Mean PV flow, mL/minute	1188	926	1500	1015	1020	1412	1523	1602	1406	1582
Mean liver mass perfusion, mL/g/minute	0.78	0.53	0.95	0.77	1.05	0.66	0.99	0.89	0.89	1.02
Viability criteria met	No	No	Yes	Yes	Yes	Yes	Yes	Yes	Yes	Yes

NOTE:

Donor WIT was defined as the interval between the systolic blood pressure <50 mm Hg and/or arterial oxygen saturation to <70% to commencing the aortic cold perfusion in the donor.

The target flow was 0.25 mL/minute/g liver tissue on the arterial side and 0.75 mL/minute/g liver tissue in the venous circuit. To achieve these flows, perfusion pressures on the device were adjusted between 30‐50 mm Hg (mean pressure) on the arterial side and 8‐10 mm Hg on the PV. The temperature was initially set at 20°C and increased incrementally to 37°C within 30 minutes of starting NMP. The target perfusate oxygen pressure was 40 kPa.

### Samples and Data Collection Protocol

Liver biopsies were taken before commencing the perfusion (t = 0), after finishing the HOPE perfusion (t = 2 hours), and on completion of NMP (t = 6 hours; Fig. 1). Biopsies were immediately either placed in formalin or snap‐frozen in liquid nitrogen for subsequent analysis. The biopsy at the end of the HOPE phase was used for ATP assessment only, and all other analyses were done on biopsies from the beginning and end of the perfusion. The perfusate was sampled every 30 minutes throughout the perfusion in both groups and analyzed immediately for blood gases or snap‐frozen and stored for later analyses. Bile produced during the perfusion was collected via a 12‐Fr silicon tube inserted into the common bile duct and weighed at 4 and 6 hours of machine perfusion in both groups. The density of bile was considered 1 g/mL, and this was normalized for liver mass.

### Assessment of Liver Function

Arterial and venous perfusates were assessed using a Cobas b 221 (Roche Diagnostics, Indianapolis, IN) point‐of‐care system blood gas analyzer. Parameters included partial pressure of oxygen and partial pressure of carbon dioxide (PCO_2_), pH, base excess, bicarbonate, O_2 _saturation, hemoglobin, hematocrit, sodium, potassium, chloride, calcium, glucose, and lactate concentrations.

Oxygen uptake was calculated during the HOPE perfusion as the difference between the oxygen inflow minus the outflow in kPa, corrected by liver weight and liters of perfusate. During NMP, the oxygen consumption was calculated from the difference between the oxygen content after the oxygenator and the return into the oxygenator in the venous circuit. Oxygen content was calculated as the sum of the free dissolved oxygen fraction to the hemoglobin‐bound oxygen fraction (equation in the Supporting Methods) as described elsewhere.^17^

The viability of the organ was assessed at the end of the perfusion by our unit’s clinical criteria, based on perfusate lactate levels falling to concentrations of <2.5 mmol/L within 6 hours, in combination with evidence of bile production, stable vascular flows, and homogeneous parenchymal perfusion.^12^

### Histopathological Assessment of Hepatocyte Injury

Menghini needle and wedge biopsies obtained prior to perfusion and at the end were fixed in formalin, processed, and embedded in paraffin. Thereafter, 4‐μm sections were cut and stained with hematoxylin‐eosin and periodic acid–Schiff (PAS). Hematoxylin‐eosin sections were semiquantitatively graded for ischemic‐type coagulative necrosis, large‐ and small‐droplet macrovesicular steatosis, and preexisting acute or chronic liver disease. The PAS‐stained sections were scored for percentage of hepatocytes depleted of glycogen, and the variation between the beginning and the end of the perfusion compared across groups. Histological assessment was conducted by an experienced liver transplant pathologist without prior knowledge of the designated perfusion category or outcome.

### Immunohistochemical Assessment of Oxidative Stress and Tissue Inflammation

Immunohistochemistry was performed on formalin‐fixed paraffin‐embedded sections to assess surrogate markers of oxidative injury and tissue inflammation.

For oxidative injury, we assessed expression of the following:

1. Uncoupling protein 2 (UCP‐2), a mitochondrial inner membrane protein that uncouples the electron transport chain from oxidative phosphorylation. Elevated uncoupling protein 2 expression is associated with increased reactive oxygen species (ROS) production.^17^, ^18^
2. 4‐hydroxynonenal (4‐HNE) as a marker of cell membrane phospholipid peroxidation.^19^

For the assessment of tissue inflammation, the following markers were analyzed:

1. Cluster of differentiation (CD)14, a lipopolysaccharide receptor, which is part of the toll‐like receptor 4 signalosome. It is essential for activation of the toll‐like receptor 4 via the recognition of ligands such as damage‐associated molecular patterns (DAMPs) known to be up‐regulated during ischemia/reperfusion injury.^18^, ^19^
2. CD11b is an integrin on the surface of leukocytes; up‐regulation on its expression indicates activation of the cells by substances including ROS.
3. Vascular cell adhesion molecule 1 (VCAM1) expression is up‐regulated on vascular endothelial cells and Kupffer cells when activated by ROS and proinflammatory cytokines during ischemia/reperfusion injury.^20^

All primary antibodies were detected using specific ImmPRESS Excel Amplified horseradish peroxidase Polymer Staining Kit specific to the respective mouse or rabbit immunoglobulin isotype. A list of primary antibodies and the dilution used is provided in the Supporting Materials.

### Immunohistochemistry Quantitation

Four pictures of each section excluding the edges were randomly selected for analysis (×400 magnification). For UCP‐2, 4‐HNE, CD14, and CD11b, a semiquantitative scoring system, the modified immunoreactive score (IRS),^21^ was obtained by multiplying the score for intensity (0, no color reaction; 1, mild reaction; 2, moderate reaction; 3, intense reaction) and distribution (0, no positive cells; 1, <10% positive cells; 2, 10%‐50% positive cells; 3, 51%‐80% positive cells; 4, >80% positive cells) to obtain a final score between 0 and 12. Change in the overall tissue expression of staining (ΔIRS) was determined by subtracting the IRS scores after 6 hours of perfusion from the score prior to perfusion: Negative values indicated a decrease, and positive values indicated an increase in the expression of the staining.

VCAM1 tissue expression was assessed by image analysis using an established system of color differentiation (ImageJ, US National Institutes of Health, Bethesda, MD), and the variation in the percentage of the positive area of staining (Δ%VCAM1) over time was compared between groups.

### *Assessment of Tissue ATP Concentration*

Quantification of ATP levels was done by homogenization of liver tissue with the concentration determined using the ATP Bioluminescent Assay kit (FLAA, Sigma‐Aldrich Inc., St. Louis, MO). More details are provided in the Supporting Materials.

### Statistical Analysis

Continuous variables were expressed as median with interquartile range (IQR) and categorical variables as absolute numbers with percentage frequencies. Comparisons between groups were performed using Fisher’s exact test for categorical variables, Mann‐Whitney U test for independent continuous variables, and Wilcoxon signed rank test for repeated measurements over time on the same sample. The statistical level of significance was set at *P *< 0.05. GraphPad Prism (version 6.04 for Windows, GraphPad Software, La Jolla, CA) software was used for all statistical analyses and graph creation.

## Results

### Donor Demographics and Discarded Liver Characteristics

Seven (70%) livers were from donation after circulatory death (DCD). The median donor age of the entire cohort was 52 years (IQR, 38‐54 years), and the body mass index was 25 kg/m^2 ^(21‐31 kg/m^2^). Median cold ischemia time (CIT) was 510 minutes (446‐682 minutes) for DCD, and 491 minutes (454‐586 minutes) for donation after brain death (DBD). The median donor risk index (DRI) was 2.2 (1.9‐2.6). There were no significant differences in donor demographics between the groups as shown in Table 1, and they had similar median DRI (NMP versus HOPE + NMP; 2.4 versus 2.5; *P* = 0.78). There was a trend toward NMP livers having shorter SCS preservation times for DCD (449 versus 682 minutes; *P* = 0.09). The detailed donor and liver data are presented in Tables 1 and 2.

**Table 2:** Donor Demographics, Liver Characteristics, and Machine Perfusion Data

	NMP (n = 5)	HOPE + NMP (n = 5)	*P* Value
Donor information			
Age, years	55 (43‐65)	54 (46‐54)	0.84
DCD livers	4 (80)	3 (60)	0.50
Sex, male	5 (100)	2 (40)	0.17
Height, cm	184 (173‐188)	179 (169‐181)	0.19
Weight, kg	78 (73‐85)	90 (87‐106)	0.39
Body mass index, kg/m^2^	23 (21‐27)	35 (30‐37)	0.08
DRI	2.4 (1.9‐2.8)	2.5 (2.2‐2.7)	0.78
UK donor liver index	3.1 (1.5‐15.7)	4.9 (4.0‐10.6)	0.89
ET DRI	2.3 (1.9‐2.9)	2.8 (2.6‐2.8)	0.80
Peak ALT, IU/L	68 (44‐137)	189 (37‐271)	0.24
Days on ventilator	3 (1‐5)	4 (2‐4)	0.89
Liver characteristics			
Liver weight, g	2208 (1998‐2218)	1935 (1838‐2060)	0.87
Donor WIT, minutes	20 (16‐22)	31 (19‐36)	0.42
CIT, minutes			
DCD	449 (423‐532)	682 (586‐708)	0.09
DBD	454 (454)	586 (490‐682)	0.57
Macrovesicular steatosis, %	0 (0‐30)	10 (1‐22)	0.98
Machine perfusion parameters			
Lactate, mmol/L			
Highest	10.3 (9.6‐10.4)	8.9 (7.8‐9.1)	0.14
Lowest	1.4 (0.6‐4.1)	0.8 (0.6‐1.1)	0.22
Last	1.6 (1.4‐5.3)	0.8 (0.6‐1.1)	0.17
Total bile production, g	18 (0‐32)	16 (0‐40)	0.82

NOTE:

Continuous variables are presented as median and IQR; dichotomous variables are presented as absolute numbers and percentages. Donor WIT was defined as the interval between the systolic blood pressure <50 mm Hg and/or arterial oxygen saturation to <70% to commencing the aortic cold perfusion in the donor.

### Vascular Flows

**Figure 2:**
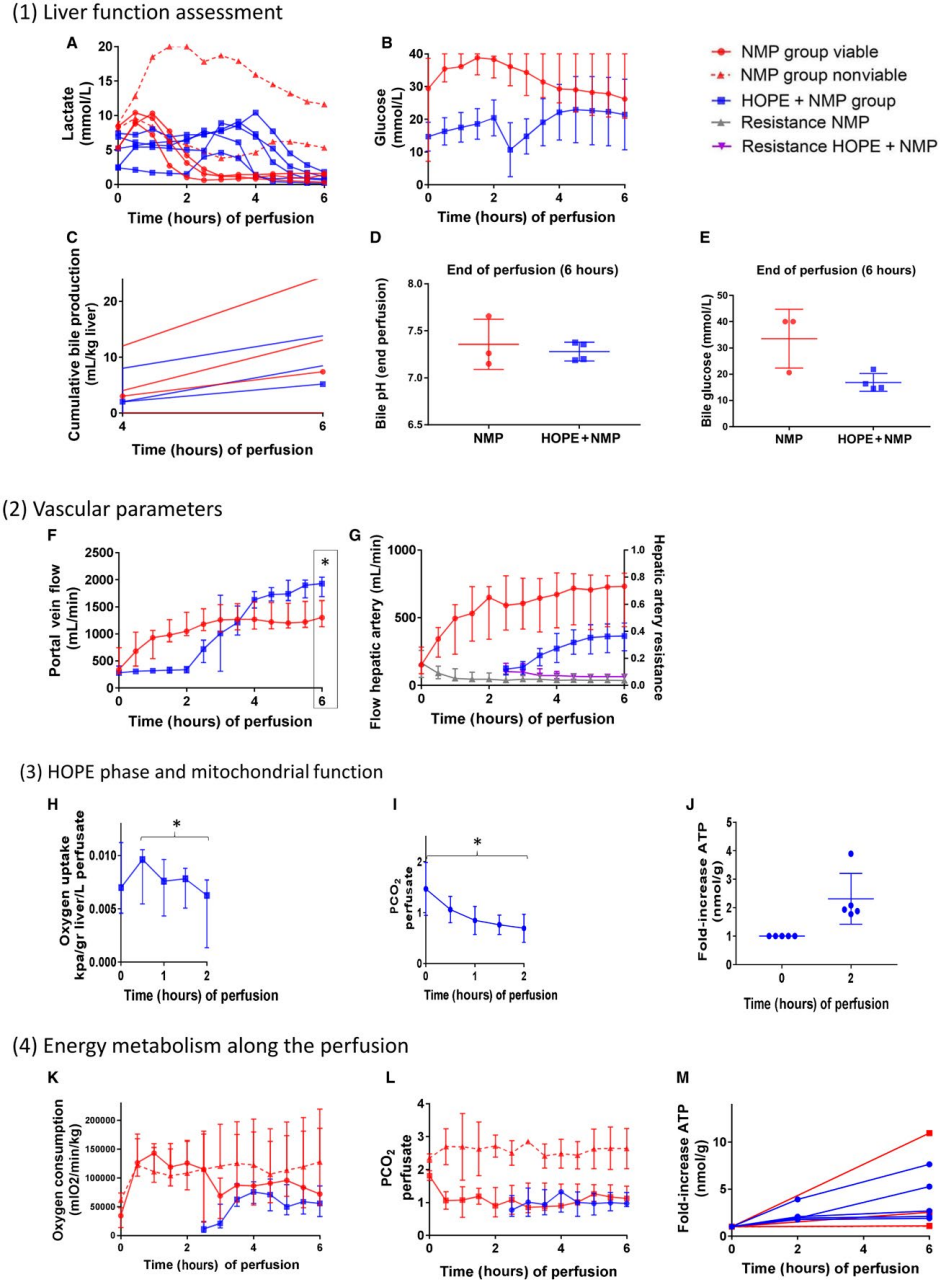
Perfusion parameters. Section 1: liver function assessment. (A) Perfusate lactate concentration (mmol/L) measured throughout the perfusion. Here, the NMP group was stratified in the viable livers (continuous red lines; 60%, n = 3) or nonviable livers (dotted red lines; 40%, n = 2). Blue lines represent the HOPE + NMP group livers. (B) Perfusate glucose concentration (mmol/L). Data are presented as median and IQR. (C) Cumulative bile production for individual livers after 4 and 6 hours of perfusion corrected for liver weight in kilograms. (D) Bile pH at 6 hours of perfusion was comparable between the study groups, as was (E) glucose. Section 2: vascular parameters of the perfusion. (F) PV flow rate in mL/minute showing that flow increased steadily in the NMP group plateauing after 2 hours. For HOPE + NMP, it remained low and stable during the HOPE phase, increased during the rewarming phase, and achieved higher rates at the end of the perfusion compared with NMP alone. (G) HA flow rate expressed in mL/minute plotted on the right *y* axis and HA vascular resistance in mm Hg/mL/minute/kg liver plotted on the left *y* axis. Despite similar resistances at the end of the perfusion, the HA flow reached higher values at the end of the perfusion in the NMP group. Section 3: HOPE phase and mitochondrial function. (H) Oxygen uptake during the HOPE phase of the HOPE + NMP group (ΔinflowO_2_‐outflowO_2_), expressed as median and IQR. (I) PCO_2_ (kPa) released in the perfusate decreased steadily throughout the hypothermic perfusion phase. (J) Tissue ATP levels (nmol/g) at time 2 hours were normalized for the concentration at time 0 and expressed as fold changes. Data are presented as median and minimum/maximum. Section 4: Energy status during perfusion. (K) Oxygen consumption during the NMP phase for both groups; NMP stratified according to viability criteria with the continuous red line representing viable and the dotted red line representing nonviable, respectively. Data are presented as median and IQR. Oxygen consumption increased sharply within the initial 60 minutes of NMP, whereas the HOPE + NMP livers presented a slower increase in oxygen consumption during the normothermic phase. (L) PCO_2_ (kPa) released in the perfusate during the NMP phase showing that figures were similar between the viable livers of the NMP group and the HOPE + NMP group. Nonviable livers from the NMP group presented with higher perfusate PCO_2_ levels throughout the entire duration of perfusion. (M) Tissue ATP levels (nmol/g) over 6 hours of perfusion. Figures at time 2 hours and 6 hours were normalized for the concentration at t = 0 and expressed as a fold increase. Levels of significance: *P < 0.05 (Wilcoxon signed rank test).

Portal flow patterns and end‐perfusion volumes differed between the groups. During the initial 60 minutes of the NMP group perfusion, the livers’ portal flow rate increased rapidly and plateaued after 180 minutes. For the HOPE + NMP group, steady low‐volume flow rates approximating 300 mL/minute were achieved during the HOPE phase. The flow rate subsequently increased for the first 2 hours of rewarming during the NMP phase. The median flow rates in the HOPE + NMP group exceeded the NMP group after 210 minutes and continued to increase until the end of the perfusion (1300 versus 1930 mL/minute; *P* = 0.03). In contrast, the arterial flows in the NMP livers increased more rapidly during the rewarming phase and remained higher throughout the perfusion (365 versus 732 mL/minute; *P* = 0.09), while the vascular resistance patterns and levels were similar. The detailed data are presented in Fig. 2 and Table 1.

### Mitochondrial Function and Oxygen Consumption

During the HOPE phase, livers demonstrated a decrease in the oxygen uptake rate and CO_2_ release in the perfusate. The oxygen uptake during the HOPE phase peaked within the initial 30 minutes (0.009 [0.005‐0.010] kPa/g liver/L perfusate) and then declined steadily (0.006 [0.001‐0.007] kPa/g liver/L perfusate) until the end of the 2‐hour perfusion (*P* = 0.04). There was a concomitant decrease in the median PCO_2_ dissolved in the perfusate from time 0 to 2 hours of hypothermic perfusion (1.3 [1.2‐1.9] to 0.7 [0.6‐0.8] kPa); *P* = 0.04). This change in mitochondrial respiration was associated with a median 1.8‐fold (range, 1.3‐3.9‐fold) increase in tissue ATP levels.

The oxygen consumption in the NMP group increased sharply within the initial 60 minutes with distinct patterns in viable compared with nonviable groups (Fig. 2). In the HOPE + NMP livers, oxygen consumption increased slower during the normothermic phase and was maximal after 2 hours. At the end of the perfusion, the oxygen consumption in viable NMP livers was similar to livers that had HOPE (*P* = 0.19).

Both groups had similar ATP stores at the end of the 6 hours of perfusion (*P* = 0.31). However, there was a significant difference in the incremental rate between viable and nonviable livers in the NMP group (NMP viable 2.5‐fold [2.4‐10.9‐fold]; NMP nonviable 1.1‐fold [1.1‐1.2‐fold]; HOPE + NMP 2.7‐fold [2.0‐6.7‐fold]; *P* = 0.05). Data are shown in Fig. 2.

### Liver Function Assessment

The lactate levels and clearance dynamics differed between the groups. In the NMP group, lactate levels peaked after 60 minutes, reached the viability criteria within 150 minutes, and subsequently remained low. In the HOPE + NMP livers, it did not change through the hypothermic phase, and it slowly increased in the rewarming phase with a subsequent rapid clearance just after 2 hours of NMP. The end‐perfusion lactate levels were similar in viable livers from the NMP group and the HOPE + NMP group (1.6 versus 0.8 mmol/L; *P* = 0.17). In the nonviable livers, lactate levels peaked after 60 minutes with minimum levels achieved by the end of the perfusion of 8.5 mmol/L. A total of 3 (60%) livers in the NMP group and 5 (100%) in the HOPE + NMP were deemed viable (*P* = 0.22). Details of the achievement of viability parameters are presented in Table 3. The 2 nonviable livers from the NMP group had the highest DRI, but this difference was not statistically significant (nonviable DRI versus viable DRI; 2.8 [2.5‐3.2] versus 2.2 [1.9‐2.2]; *P* = 0.13).

**Table 3:** Viability Criteria Achievement by the Livers in Each Group

Criteria	NMP (n = 5)	HOPE + NMP (n = 5)
Lactate clearance (≤2.5 mmol/L)	3 (60)	5 (100)
pH >7.3 perfusate	2 (40)	2 (40)
Glucose metabolism	3 (60)	4 (80)
HA flow (>150 mL/minute)	5 (100)	5 (100)
PV flow (>500 mL/minute)	5 (100)	5 (100)
Homogeneous perfusion/soft parenchyma	5 (100)	5 (100)
Bile production	3 (60)	3 (60)
Viable liver	3 (60)	5 (100)

NOTE:

Data are given as n (%).

The glucose levels slowly increased within the initial 120 minutes of perfusion in the NMP group, followed by slow clearance until the end of the perfusion. In the HOPE + NMP group, they slowly increased through the hypothermic perfusion, followed by a drop (related to the perfusate fluid replacement) with a subsequent slow increase, and then decreased, reaching similar figures to the NMP group at the end of the perfusion (26 [20‐40] versus 16 [1‐17] mmol/L; *P* = 0.22).

Bile production at 6 hours of the perfusion was similar between both groups (NMP versus HOPE + NMP; 7.4 versus 5.2 mL/kg liver; *P* = 0.82). Bile pH (HOPE + NMP versus NMP; 7.3 [7.2‐7.4] versus 7.3 [7.2‐7.5]; *P *> 0.99) and bile glucose (HOPE + NMP versus NMP; 13 [9‐16] versus 21 [14‐27]) were comparable between the groups at the end of the perfusion. Data are represented in Fig. 2.

### Histological Assessment of the Livers

**Figure 3:**
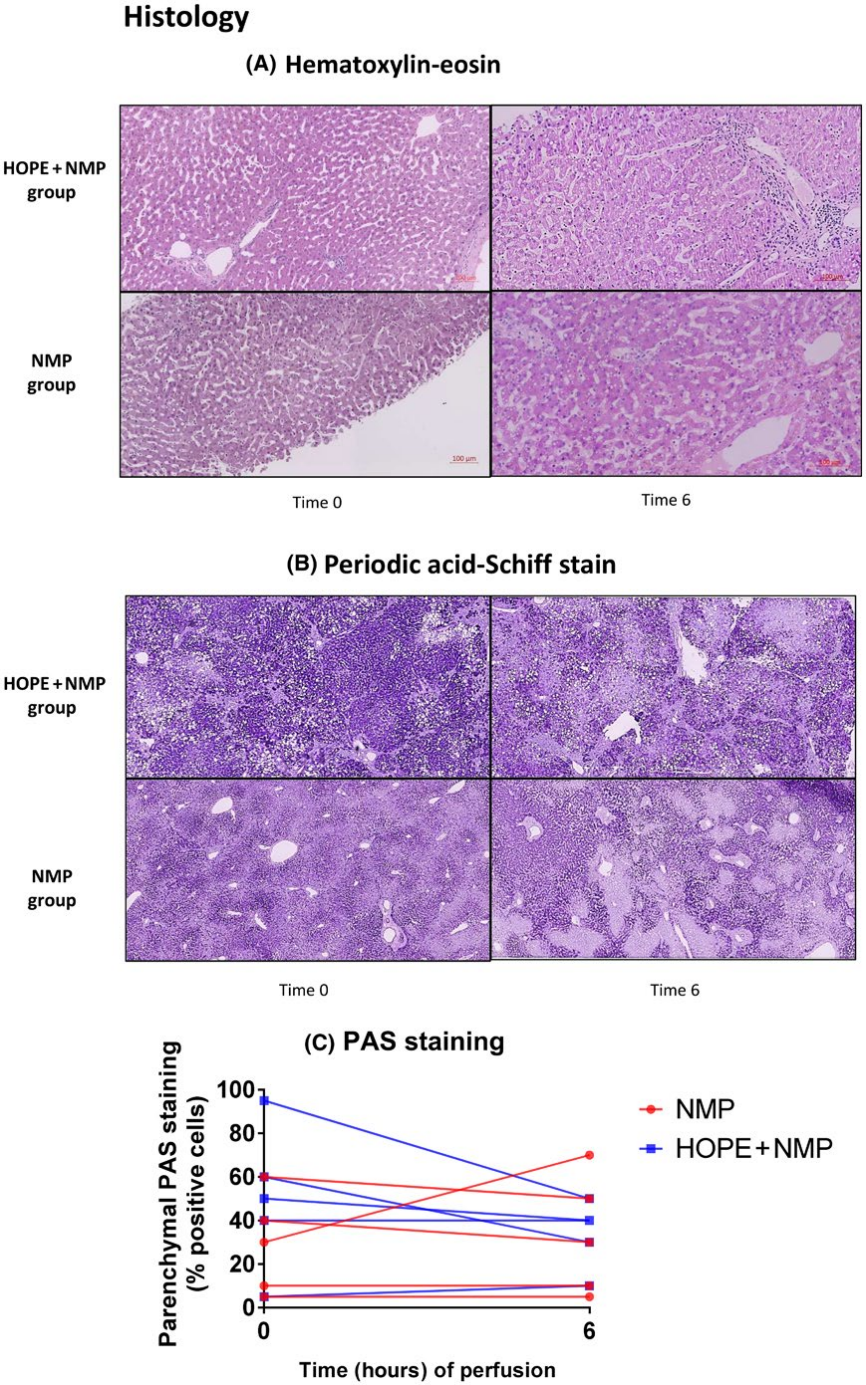
Liver histology before and after machine perfusion. (A) Hematoxylin‐eosin and the (B) PAS staining at the beginning (t = 0) and end of the perfusion (t = 6 hours). No observable histological differences were seen between the 2 groups prior to or at the end of perfusion. There was no evidence of perfusion‐related ischemic coagulative necrosis developed in any liver during perfusion. (C) The percentage of areas of glycogen deposition (parenchymal PAS staining) from start to end of the perfusion was also similar in both groups.

Routine histological assessment showed no observable differences between the groups on the biopsy prior to the perfusion. At the end of the perfusion, none of the livers presented with areas of parenchymal necrosis, congestion, or cytoplasmic vacuolization. The median large‐droplet macrovesicular steatosis percentage was not significantly different between the groups (NMP versus HOPE + NMP; 0% [0%‐30%] versus 10% [1%‐22%]; *P* = 0.98), and only 1 liver from each group presented with steatohepatitis. The levels of hepatocyte glycogen depletion from the start to the end of the perfusion period were similar in both groups (NMP versus HOPE + NMP; 0% [–20/10] versus 10% [2/32]; *P* = 0.22) with a positive value reflecting an increase in the percentage of hepatocytes depleted of glycogen. Data are presented in Fig. 3.

## Immunohistochemical Assessment of Oxidative Stress and Tissue Inflammation

**Figure 4:**
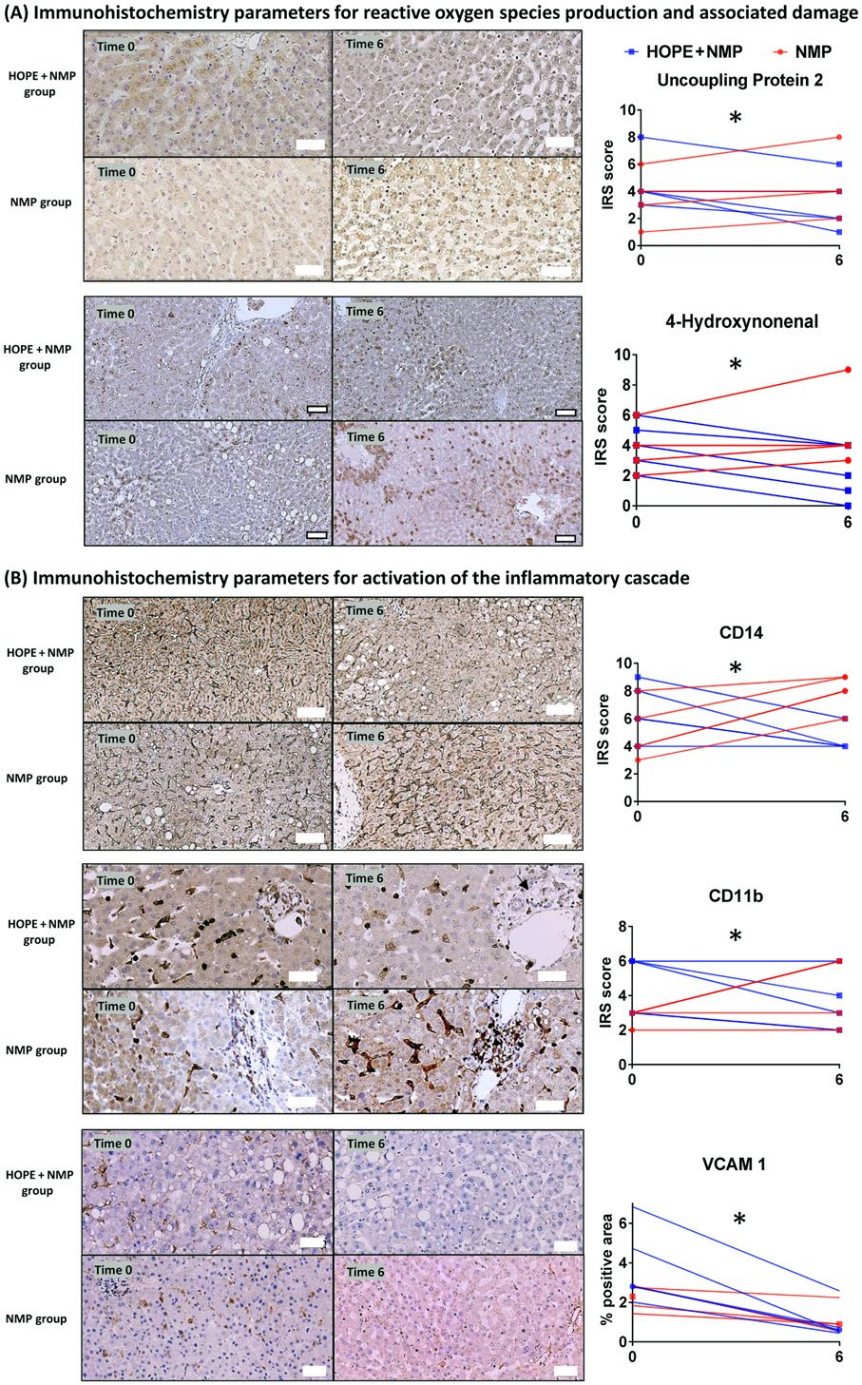
Immunohistochemical analysis for markers of ROS‐mediated damage and tissue inflammation. The upper row of each panel shows representative images for the HOPE + NMP group, and the lower images show representative images for the NMP group prior to and at the end of perfusion (t = 0 and t = 6 hours). (A) There was a consistent reduction in expression of markers for oxidative injury in the HOPE + NMP group as reflected by the IRS. In contrast, an opposite trend was seen for the NMP livers over the course of the perfusion. (B) Markers indicative of activation of the inflammatory cascade also showed reduced expression by the end of perfusion in the HOPE + NMP group. Although VCAM1 expression decreased in both groups, it trended toward being more pronounced in livers that had undergone HOPE + NMP. Bold outside border white squares at the bottom of figures represents 50 µm, and no border white squares represent 100‐µm scaling. Levels of significance: *P < 0.05 (Wilcoxon signed rank test).

Immunohistochemical analysis showed that livers subjected to NMP alone expressed higher levels of tissue markers of oxidative injury and inflammation compared with those subjected to HOPE + NMP by the end of the perfusion run. Livers that had HOPE showed a significant reduction in expression of markers associated with mitochondrial ROS production (UCP‐2; NMP ΔIRS versus HOPE + NMP ΔIRS; 1 versus –2; *P* = 0.02) and oxidative stress–mediated cellular injury (4‐HNE) expression reflecting lipid peroxidation (NMP ΔIRS versus HOPE + NMP ΔIRS; 1 versus –2; *P* = 0.008). Both proteins were present predominantly in hepatocytes. Similarly, they showed decreased expression of CD14 at the end of the perfusion compared with commencement. A statistically significant opposite trend was seen for the livers perfused using NMP only (NMP ΔIRS versus HOPE + NMP ΔIRS; 3 versus –2; *P* = 0.008). The intensity of the tissue inflammatory response was shown by different patterns of nonparenchymal cell staining for CD11b, which was present on polymorphonuclear leukocytes (neutrophils and monocytes/macrophages). There was a significant difference in its level of expression between the 2 groups throughout the perfusion (NMP ΔIRS versus HOPE + NMP ΔIRS; 3 versus –1; *P* = 0.02), suggesting a potential beneficial effect of the combined protocol. Intrahepatic endothelial cells play a key role in reperfusion injury by promoting leukocyte adhesion and retention. To evaluate the (cytokine‐mediated) activation of endothelial cells, the percentages positive areas of VCAM1 staining were determined. VCAM1 expression decreased over time in both groups, but this was more pronounced in livers that had undergone HOPE (NMP Δ%VCAM1 versus HOPE + NMP Δ%VCAM1; –0.5 versus –2.2; *P* = 0.05). Data are presented in Fig. 4.

## Discussion

Machine perfusion was developed to minimize damage during organ preservation, and early clinical experience proved its superiority over SCS and positive impact on extended criteria liver utilization.^8^, ^12^, ^13^, ^22^, ^23^ The 2 leading perfusion strategies, hypothermic and normothermic technique, have previously been viewed as diverse, or even competing, approaches with each having advantages and disadvantages.^24^ In the present study, we compared a protocol that combined the 2 methods to evaluate whether the combination would provide the potential benefits of both. We found that initial HOPE perfusion promoted recovery of mitochondrial function, increased ATP energy stores, and lowered tissue injury during subsequent NMP. Normothermic perfusion allowing viability assessment is seen by our group as an essential aspect of patient safety for transplantation of the highest‐risk and/or currently unused livers. The combination of HOPE with NMP translated into the increased rate of functional recovery of livers pretreated with HOPE compared with livers subjected to NMP alone.

Our team became an early adopter of the NMP technique, and we observed that a proportion of livers failed to recover their function.^12^ Those poor‐quality organs, often exposed to prolonged CITs, were unable to maintain vascular flows, clear lactate, or produce any bile. With the mounting evidence of the protective mechanism of HOPE, we designed the combined perfusion to assess if this intervention would yield a superior functional recovery for the highest‐risk discarded livers.^7^, ^22^, ^24^, ^25^ Our key objective in designing this experiment was to imitate the real‐life situation where clinical decisions are required regarding transplantability of a clearly suboptimal liver upon its arrival to the transplant center.

Although the use of HOPE with subsequent NMP was reported by the Groningen group previously, their experiment was aimed at assessing benefit of HOPE compared with SCS and used the NMP phase to simulate in vivo reperfusion.^26^ In this scenario, the overall perfusion time on the device at the point of the functional assessment was beyond the certified scope of 6‐hour use, limiting its value for translation toward clinical adoption.^26^, ^27^

Our study represents a novel approach and shows unique data, as it assesses the viability of human donor livers using clinically validated criteria.^12^, ^28^, ^29^ Previous rodent models of ECD livers showed that a short period of HOPE was able to promote recovery of mitochondrial function, optimizing oxygen utilization and recovering cellular ATP energy stores.^25^, ^30^ HOPE was suggested to promote metabolism of the succinate accumulated during the ischemic period under hypothermic conditions, subsequently diminishing the reversal flow of electrons from the mitochondria and the production of ROS during the rewarming.^31^

Although there has been extensive mechanistic research using animal models showing a down‐regulation in the tissue inflammatory responses, including activation of endothelial and resident immune cells of the liver, the evidence from human livers, especially those from the highest‐risk donor pool, is very scarce.^18^, ^24^ In order to assess the extent of the injury developed during rewarming on the machine, we analyzed numerous markers of nonparenchymal cell activation. In accordance with previous observations, we confirmed the putative benefits of HOPE, in comparison to negligible changes or even an up‐regulation of detrimental processes observed during NMP perfusion alone.^24^, ^31^ We have shown changes in the mitochondrial respiratory rate during the HOPE phase of the combined perfusion group that correlated with lower activation of the inflammatory cascade and oxidative injury at the end of 6 hours of machine perfusion in comparison with NMP alone. Replacement of the perfusate, which may reduce toxic metabolites and detritus accumulated during SCS, and recirculating perfusate may also further enhance reduction of the tissue inflammatory response and injury in the HOPE + NMP group. The lack of the liver flush following the HOPE phase might alter the perfusion fluid constitution for the normothermic perfusion in the HOPE + NMP group. However, analyses of these minimal differences between the groups were beyond the scope of the present study.^32^, ^33^

**Figure 5:**
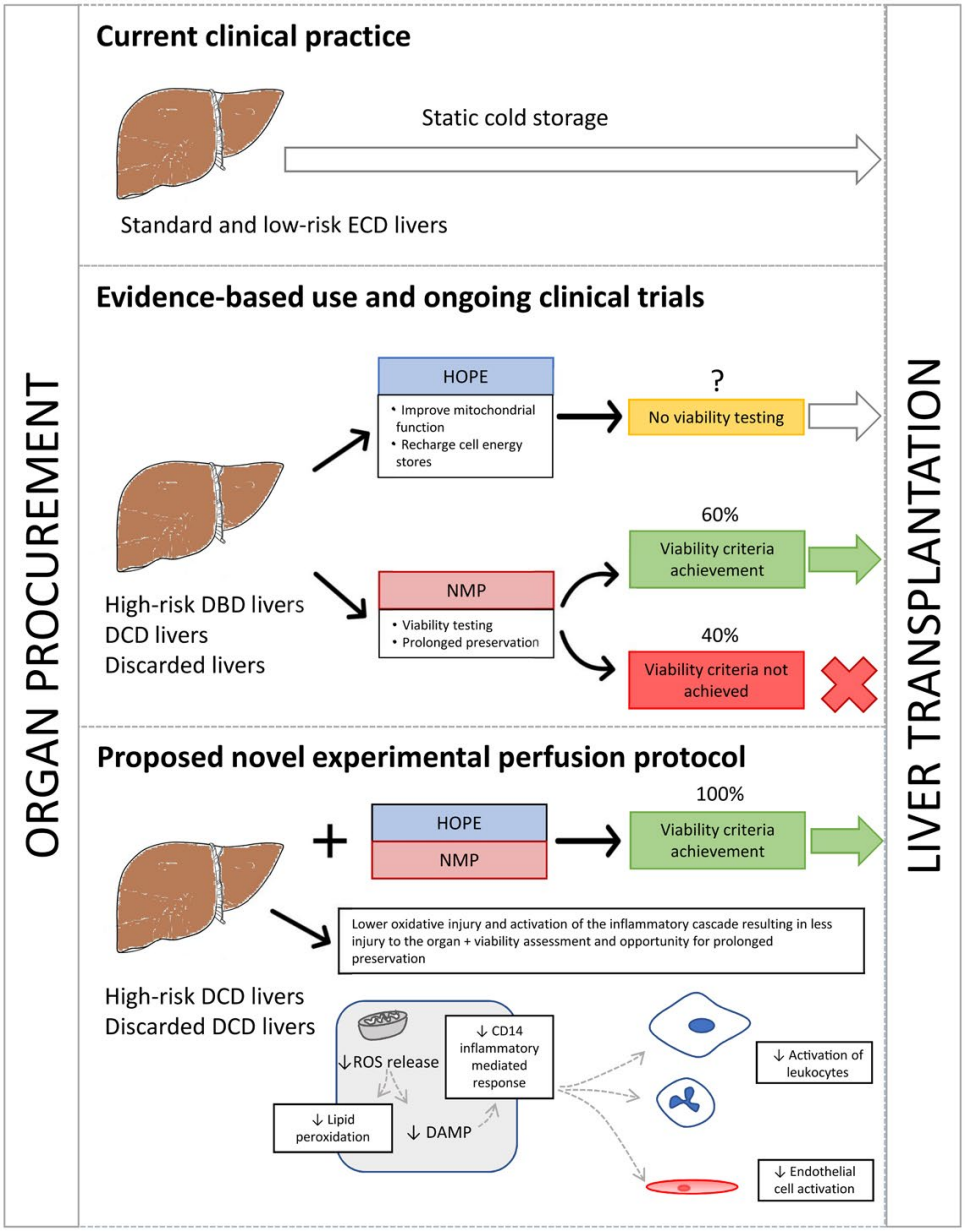
Diagrammatic summary of the findings and proposal for the use of a combined protocol of ex situ machine perfusion encompassing HOPE and NMP for the recovery of ECD livers. The top panel of the diagram illustrates current clinical practice for standard or low‐risk extended criteria organs, which are preserved using the traditional SCS. The middle panel shows the potentially beneficial effects of each machine perfusion protocol. HOPE perfusion does not permit objective organ viability assessment, which limits its potential use for high‐risk ECD livers. NMP potentially allows viability assessment prior to transplantation, making this option extremely valuable for high‐risk ECD livers. In fact, a significant proportion of these high‐risk organs do not achieve our viability criteria when submitted to NMP alone. The bottom panel summarizes the outcome and possible mechanistic benefits of the combined protocol of HOPE + NMP. This newly developed protocol was shown to derive individual benefits of both techniques. This is reflected by demonstrating a higher rescue of high‐risk ECD livers in the HOPE + NMP group than NMP alone.

NMP provides a near‐physiological environment for the liver by supplying oxygen and nutrients at normothermic condition, enabling the organ functional assessment. Our previous work on end‐ischemic NMP of declined human livers allowed us to define the viability criteria employed for transplantation of discarded human livers.^12^, ^28^ Setting the achievement of viability criteria already used for clinical transplantation as an endpoint is an important strength of this study. However, we appreciate that a consensus does not yet exist regarding optimal parameters for organ viability assessment during NMP.^17^, ^26^, ^34^, ^35^ Lactate clearance to levels lower than 2.5 mmol/L is a principal parameter for viability criteria used at our unit. Although this marker is seen in our clinical practice as a highly sensitive and specific parameter to predict early graft failure due to primary nonfunction, Watson et al. recently advocated transaminase levels in the perfusate and bile quality assessment, by the pH and glucose content, to be superior in terms of predicting late graft loss due to biliary complications.^35^ This is a pertinent point because the previous clinical studies have demonstrated the key advantage of HOPE perfusion being preventative of nonanastomotic biliary strictures in DCD livers, and these organs might benefit most from the combined protocol.^7^, ^32^ The presented data showed improved viability also in DBD livers. Despite the limited number of perfusions included in our study, all assays from the HOPE + NMP group consistently showed cytoprotective effects and superior parameters, including clinical viability criteria (a diagrammatic summary is presented in Fig. 5). This was demonstrated by all HOPE + NMP organs achieving the viability criteria, whereas 40% (2/5) of the organs perfused by NMP alone failed to recover enough function to be deemed transplantable.

The use of an acellular hemoglobin‐based oxygen carrier was shown by our group and others to be a suitable alternative to the use of packed red cells in the context of NMP.^16^, ^36^ An acellular oxygen carrier–based fluid could be potentially used throughout the whole perfusion, avoiding any change of perfusate. Nevertheless, in logistic terms, the disconnection of the liver from the perfusion circuit for approximately 20 minutes, which is necessary to exchange the device perfusate, seemingly did not cause any measurable harm to the organs temporarily placed on ice. HOPE perfusion via PV only was previously shown to provide sufficient oxygenation for the entire liver, including the extrahepatic biliary tree, without any need for manipulation of the HA at this stage.^7^, ^24^, ^37^

The feasibility of combining variant perfusion techniques has been suggested before, though the advantages, caveats, and logistics aspects of different combinations are yet to be seen.^38^ The current study is the first to show a combined protocol of HOPE + NMP that might be easily adopted to clinical practice. A short‐term HOPE phase optimizes mitochondrial oxidative function, decreasing tissue oxidative injury and the downstream activation of the inflammatory cascade during reperfusion. Thus, HOPE mitigates ischaemia/reperfusion injury during NMP. Those factors together might be essential to minimize any damage and improve functional recovery in the highest‐risk ECD livers damaged by cold ischemia during the SCS prior to the end‐ischemic machine perfusion. This approach differs from NMP preservation when the organs are exposed only to a very short period of ischemia.^23^ The Liver Assist device enables variation of perfusion strategies in temperatures ranging from 10°C to 37°C within the device‐certified 6‐hour timeframe. The presented study used 2 hours of HOPE and 4 hours of NMP, durations previously recommended by other groups, allowing for the use of a single perfusion kit and rendering the combined perfusion cost‐effective.^23^, ^26^

The present study has some caveats. It is not a transplant model because this would not be clinically possible in the United Kingdom at this early investigative stage. Although the reperfusion injury could be simulated by a subsequent NMP with whole blood, we opted to study the organ functional recovery only, and we have applied our current viability criteria to define its potential transplantability. Those criteria have been used by our team in clinical trials to safely transplant discarded human livers, allowing us to compare the findings and predict transplantability and clinical relevance of our preclinical proof of concept studies.^12^, ^29^ The use of discarded human livers for research confers the advantage of eliminating between‐species variability, which is a limitation for animal models where the extrapolation of results to humans is questionable. The scarcity and intrinsic heterogeneity among discarded human livers, however, makes it difficult to achieve perfectly matched study groups.

Using an acellular oxygen carrier fluid also precludes any direct comparison of our data with frequently published machine perfusion studies’ endpoints, including perfusate transaminases.^23^, ^34^, ^35^ The free hemoglobin concentration in the Hemopure‐based perfusate exceeded the maximum hemolytic index tolerance for our hospital clinical laboratory assessment, and we were unable to measure those subsequently by alternative methods from rethawed frozen samples. As a similar perfusion fluid has already been used for clinical perfusions, we expect that it might be soon widely adopted and our results validated by others.

In conclusion, this proof of concept study demonstrated that the combination of sequential HOPE and NMP is not only feasible, but that it may potentially improve the functional recovery of high‐risk ECD livers compared with NMP alone. Although we do not suggest that this is an optimized protocol, this novel approach might be particularly beneficial for DCD organs. Further studies are needed to explore whether the combined protocol confers other benefits, such as the reduced biliary complications of HOPE or the safety of prolonged perfusions of NMP.
