# Supplementary material for: Combined Hypothermic and Normothermic Machine Perfusion Improves Functional Recovery of Extended Criteria Donor Livers
Source: Liver Transpl. 2018 Dec 4;24(12):1699–715. doi: 10.1002/lt.25315 (PMC6588092; doi:10.1002/lt.25315)
Supplement: Supplementary file 1 [file LT-24-1699-s001.docx]

**2. Supplementary Table S1**

| **Table S1: Normothermic machine perfusion fluid constitution** |
| --- |
| 1000mL (4 units) acellular oxygen hemoglobin carrier Hemopure  (hemoglobin glutamer-250-bovine; HBOC-201, Hemoglobin Oxygen Therapeutics LLC, Cambridge, MA) |
| 1000mL 5% w/v human albumin solution (Alburex 5, CSL Behring GmbH, Germany) |
| 10,000IU heparin (Wockhardt, UK) |
| 30mL sodium bicarbonate 8.4% (B. Braun Medical Limited, UK) |
| 10mL calcium gluconate 10% |
| 500mg vancomycin (Wockhardt, UK) |
| 60mg gentamicin (Cidomycin, Sanofi, UK) |
| 50mL 10% v/v Aminoplasmal (B.Braun Medical Limited, UK) |
| 0.2mL Cernevit (Baxter Healthcare Ltd., UK) |
| 0.1mg phytomenadione (Konakion, Roche Products Ltd, UK) |
| Epoprostenol (Flolan, GlaxoSmithKline, UK, 2µg/mL) continuous infusion commencing at 4mL/hour |
| **Abbreviations:** MA- Massachusetts; UK- United Kingdom. |
